# Genome Mining and Synthetic Biology in Marine Natural Product Discovery

**DOI:** 10.3390/md18120615

**Published:** 2020-12-03

**Authors:** Maria Costantini

**Affiliations:** Department of Marine Biotechnology, Stazione Zoologica Anton Dohrn, Villa Comunale, 80121 Napoli, Italy; maria.costantini@szn.it; Tel.: +39-081-583-3285

Oceans cover nearly 70% of the earth’s surface and host a huge ecological, chemical, and biological diversity. The natural conditions of the sea favor, in marine organisms, the production of a large variety of novel molecules with great pharmaceutical potential. Marine organisms are unique in their structural and functional features compared to terrestrial ones [1,2]. Innovation in this field is very rapid, as revealed by the funding of several Seventh Framework Programme (FP7) and Horizon 2020 projects under the topic “Blue Growth”. The major parts of these projects have the common final goal of meeting the urgent need to discover new drug entities to counteract the increased incidence of severe diseases (such as cancer) and the reduced efficacy of existing drugs [3]. 

For this reason, the application of molecular biology techniques in biotechnological field is very important. Driven by the rapidly-decreasing cost and increasing throughput of DNA-sequencing technologies, significant progress in genomics has renewed interest in the discovery of natural products. Rapidly-expanding genomic and metagenomic datasets reveal a vast number of biosynthetic gene clusters (BGCs) in nature, which are predicted to encode novel biomedically-relevant molecules. This ‘top-down’ discovery approach can only provide access to a small fraction of biosynthetic compounds, given that the majority of microorganisms cannot be isolated or cultured. Furthermore, even in culturable organisms, the encoded secondary metabolites of many biosynthetic gene clusters are unknown. This may be due to strong down-regulation of product biosynthesis at the transcriptional, translational, and/or post-translational levels in the absence of the right activating cues in the laboratory. Alternatively, secondary metabolites that are produced at very low yields may escape detection and characterization. Natural product discovery, which used to be predominantly an analytical chemistry problem, has become a challenge in genomics and molecular biology with respect to how to manipulate relevant genes and sequences to produce the desired encoded metabolites.

In recent years, marine genomics has grown rapidly, helped by the large amount of information that is are becoming available to the international scientific community. Taking into account the current excitement in the field of marine biotechnology, we have assembled this Special Issue entitled “Genome Mining and Synthetic Biology in Marine Natural Product Discovery”. The aim of this Special Issue is to assess the impact of these molecular approaches on the discovery of bioactive compounds from marine organisms. The term “genome mining” is used to identify all bioinformatic investigation aimed at detecting the biosynthetic pathways of bioactive natural products and their possible functional and chemical interactions [4]. Genome mining includes the identification of BGCs of previously-uncharacterized natural products within the genomes of sequenced organisms, sequence analysis of the enzymes encoded by these gene clusters, and the experimental identification of the products of the gene clusters [5].

This special issue includes one review article and four original studies on the application of genome mining and synthetic biology in promoting marine natural products for biotechnological applications. 

An up-to-date view on the importance of genome mining to identify new natural products is provided in the review by Albarano et al. [6] In the first part of this review, the significance of genome mining and other associated techniques is discussed, as well as their possible advantages and disadvantages. One of the strengths of using genome mining is the detection of a large amount of bioactive natural compounds. Genome mining has the advantages of being relatively cheap and easy to apply in the laboratory and not requiring particular skills and/or experience by the operators. The weaknesses of this bioinformatic approach are that: (1) only known biosynthetic gene clusters can be identified and (2) it is not possible to predict the biotechnological potential of the natural products under analysis, as well as to formulate their chemical structures. In addition, the authors include not only marine organisms but also terrestrial ones, considering that molecular approaches were only recently applied to the discovery of bioactive compounds from the sea. 

A very important point arising from this review is that the available literature on genome mining mainly concerns bacteria, from which the majority of new natural drugs are isolated. In fact, Gao et al. [7] report on the cloning, recombinant expression, and characterization of the ulvan lyase (able to degrade ulvan to oligosaccharides) gene, *ALT3695*, from marine bacterium *Alteromonas* sp. A321. Its amino acid sequence exhibits low sequence identity with the polysaccharide lyase family 25 (PL25) ulvan lyases from *Pseudoalteromonas* sp. PLSV (64.14%), *Alteromonas* sp. LOR (62.68%), and *Nonlabens ulvanivorans* PLR (57.37%). Ulvan is a type of cell wall polysaccharide, extracted from marine green seaweed (belonging to the genera *Ulva* and *Enteromorpha*), with a great biotechnological potential thanks to its various pharmacological properties including antioxidant, anticoagulant, antitumor, antihyperlipidemic, and antiviral activities. Furthermore, ulvan-derived oligosaccharides has applications in the functional food and pharmaceutical industries. ALT3695 exhibits excellent stability and substrate affinity, showing that it could be used for the preparation of ulvan oligosaccharides. 

Other important sources of natural drugs are the fungi. Examples of such drugs are penicillin, cephalosporin, ergotrate, and the statins. Guided by bioinformatic analysis of the genomic sequence of a marine-derived fungal strain of *Penicillium dipodomyis* YJ-11, Yu et al. [8] report for the first time on the application of global regulator LaeA in *P. dipodomyis* (PdLaeA), aiming to the increase the secondary metabolite. In fact, this regulator is able to influence fungi in many aspects of their life, such as increasing or reducing secondary metabolite production, activating cryptic gene clusters, asexual and sexual differentiation, sporulation, and pigmentation. The overexpression of the PdLaeA gene in *P. dipodomyis* YJ-11 lead to the discovery of two new compounds, 10,11-dihydrobislongiquinolide and 10,11,16,17-tetrahydrobislongiquinolide, together with four known sorbicillin analogues. 

Microalgae are eukaryotic photosynthetic microorganisms, which can be cultivated in large quantities, and for this reason they are receiving increasing attention from biotechnologists. They are able to produce a wide diversity of species and natural products with pharmaceutical, nutraceutical, and cosmeceutical applications. Riccio et al. [9] investigated the presence of genes involved in monogalactosyldiacylglycerols (MGDGs) and sulfoglycolipid synthesis in microalgae, considering all the microalgal transcriptomes and metatranscriptomes available from the Marine Microbial Eukaryote Transcriptome Sequencing Project (MMETSP) and the recent Tara Oceans and Global Ocean sampling expedition. In particular, the presence of MGDG synthase (MGD), UDP-sulfoquinovose synthase (SQD1), and sulfoquinovosyltransferase (SQD2) were identified in different microalgal classes. Not all the sequences are expressed within each taxa analyzed, suggesting the absence of a specific gene. This study prompted the discovery of new bioactive species with new insights into enzyme discovery from microalgae for biotechnological applications.

Zhu et al. [10] performed research on the genome of *Thraustochytriidae* sp. SZU445, a single-celled eukaryotic marine protist belonging to the class Labyrinthulomycetes. Using a third-generation sequencing platform, fatty acid synthesis pathways, specifically for docosapentaenoic acid (DHA) docosahexaenoic acid (DPA) were identified, predicting and analyzing the Gene Ontology (GO) and Kyoto Encyclopedia of Genes and Genomes (KEGG) databases. This represents a very important step for the scientific community because these marine protists can be cultivated to yield high biomasses containing substantial quantities of lipids abundant in polyunsaturated fatty acids (PUFAs), and so also accumulating large amounts of DHA. 

Even though these new molecular approaches need to be ecxpanded, this Special Issue of Marine Drugs provides an interesting panorama of current research on genome mining and other associated experimental techniques. As Guest Editor, I trust that this collection will help the scientific community to inspire the next generation of marine biotechnologies. In fact, molecular and bioinformatic approaches permit us to overcome the over-utilization of marine resources and the use of destructive collection practices, and to apply a more environmentally-friendly approach to drug discovery 

## References

[1] Jimeno J., Faircloth G., Fernandez Sousa-Faro J.M., Scheuer P., Rinehart K. (2004). New marine derived anticancer therapeutics. A journey from the sea to clinical trials. Mar. Drugs.

[2] Kijjoa A., Sawangwong P. (2004). Drugs and cosmetics from the sea. Mar. Drugs.

[3] Romano G., Costantini M., Sansone C., Lauritano C., Ruocco N., Ianora A. (2017). Marine microorganisms as a promising and sustainable source of bioactive molecules. Mar. Environ. Res..

[4] Ziemert N., Alanjary M., Weber T. (2016). The evolution of genome mining in microbes—A review. Nat. Prod. Rep..

[5] Trivella D.B.B., De Felicio R. (2018). The Tripod for Bacterial Natural Product Discovery: Genome Mining, Silent Pathway Induction, and Mass Spectrometry-Based Molecular Networking. mSystems.

[6] Albarano L., Esposito R., Ruocco N., Costantini M. (2020). Genome mining as new challenge in natural products discovery. Mar. Drugs.

[7] Gao J., Du C., Chi Y., Zuo S., Ye H., Wang P. (2019). Cloning, Expression, and Characterization of a New PL25 Family Ulvan Lyase from Marine Bacterium *Alteromonas* sp. A321. Mar. Drugs.

[8] Yu J., Han H., Zhang X., Ma C., Sun C., Che Q., Gu Q., Zhu T., Zhang G., Li D. (2019). Discovery of Two New Sorbicillinoids by Overexpression of the Global Regulator LaeA in a Marine-Derived Fungus *Penicillium dipodomyis* YJ-11. Mar. Drugs.

[9] Riccio G., De Luca D., Lauritano C. (2020). Monogalactosyldiacylglycerol and Sulfolipid Synthesis in Microalgae. Mar. Drugs.

[10] Zhu X., Li S., Liu L., Li S., Luo Y., Lv C., Wang B., Cheng C.H.K., Chen H., Yang X. (2020). Genome Sequencing and Analysis of *Thraustochytriidae* sp. SZU445 Provides Novel Insights into the Polyunsaturated Fatty Acid Biosynthesis Pathway. Mar. Drugs.

